# Synergistic Drug‐Loaded Shear‐Thinning Star Polymer Hydrogel Facilitates Gastrointestinal Lesion Resection and Promotes Wound Healing

**DOI:** 10.1002/advs.202309586

**Published:** 2024-04-30

**Authors:** Yue Zhang, Dongtian Miao, Mingli Su, Yinxiang Tang, Minghong Zhou, Yang Yu, Xuefeng Guo, Dingcai Wu

**Affiliations:** ^1^ PCFM Lab School of Chemistry Sun Yat‐sen University Guangzhou 510006 P. R. China; ^2^ Department of General Surgery (Endoscopic Surgery) Guangdong Provincial Key Laboratory of Colorectal and Pelvic Floor Diseases Biomedical Innovation Center Key Laboratory of Human Microbiome and Chronic Diseases (Sun Yat‐sen University) Ministry of Education Guangzhou 510655 P. R. China; ^3^ The Sixth Affiliated Hospital Sun Yat‐sen University Guangzhou 510655 P. R. China; ^4^ Medical Research Institute Guangdong Provincial People's Hospital (Guangdong Academy of Medical Sciences) Southern Medical University Guangzhou 510080 P. R. China; ^5^ Department of General Surgery (Colorectal Surgery) Guangdong Institute of Gastroenterology Biomedical Innovation Center Guangdong Provincial Key Laboratory of Colorectal and Pelvic Floor Diseases The Sixth Affiliated Hospital Sun Yat‐sen University Guangzhou 510655 P. R. China

**Keywords:** drug loading/release, multi‐arm star polymer, shear‐thinning, submucosal injection materials

## Abstract

Easy injection, long‐lasting barrier, and drug loading are the critical properties of submucosal injection materials for endoscopic surgery. However, conventional injectable polymers face challenges in simultaneously attaining these properties due to the inherent conflict between injectability and in situ stability. Here, a multi‐arm star polymer hydrogel (denoted as βCP hydrogel) with long‐lasting submucosal barrier (exceeding 120 min), rapid hemostasis, and sustained antibacterial properties is successfully developed by grafting poly(oligo(ethylene glycol) methyl ether methacrylate) (PEGMA) side‐chains from β‐CD via atom transfer radical polymerization (ATRP). During the onset of shearing, βCP hydrogel experiences the unwinding of polymer side‐chains between neighboring star polymers, which facilitates the process of endoscopic injectability. After submucosal injection, βCP hydrogel undergoes the winding of polymer side‐chains, thereby establishing a long‐lasting barrier cushion. Meanwhile, owing to its distinctive structures with a hydrophobic inner cavity and an outer layer of hydrophilic polymer side‐chains, βCP hydrogel enables simultaneous loading and on‐demand release of diverse categories of drugs. This unique performance can adapt to the diverse demands during different stages of wound healing in a porcine endoscopic surgery model. These results indicate an appealing prospect for new application of star polymers as a good submucosal injection material in endoscopic treatments.

## Graphical abstract

Shear‐thinning star polymer hydrogel from β‐CD (βCP hydrogel) synthesized by ATRP exhibits good injectability, excellent in situ stability, and synergistic drug‐loaded capability, showing an appealing application prospect for submucosal injection material in endoscopic treatments.

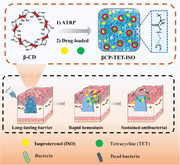

## 1 Introduction

Gastrointestinal endoscopy is widely recognized as a highly efficient and direct approach for diagnosing gastrointestinal diseases, as well as serving as the primary treatment for early gastrointestinal lesions.^[^ ^1^, ^2^ ^]^ By means of endoscopic procedures, it becomes feasible to eliminate polyps or early tumors that are confined to the mucosal layer of the gastrointestinal tract, thereby substantially enhancing the prognosis of patients with gastrointestinal tumors. Nevertheless, it is crucial to acknowledge that the thin gastrointestinal wall is divided into four layers, namely the mucosa, submucosa, muscularis propria, and serosal layer.^[^ ^3^ ^]^ As a result, during the process of resecting mucosal lesions, there is a propensity for complications such as injury to muscular layer, postoperative bleeding, and perforation.^[^ ^4^, ^5^ ^]^ In addition, the presence of a substantial quantity of bacteria and the continuous passage of contents in the gastrointestinal tract present significant obstacles to the healing of gastrointestinal wound. Consequently, the attainment of precise resection of mucosal lesions and accelerated wound healing has emerged as a crucial impediment in endoscopic treatment.^[^ ^6^, ^7^ ^]^

Injectable materials have the potential to establish a physical barrier between the lesion mucosa and muscularis propria, thereby facilitating the endoscopic resection of mucosal lesions and safeguarding the underlying deep muscle layer. Small molecule weight injectable materials like normal saline (NS) and glycerin would quickly dissipated after injection, resulting in unsatisfactory barrier effects.^[^ ^8^ ^]^ Although some high molecule weight injectable materials such as sodium hyaluronate (SH) solution, conventional shear‐thinning hydrogels, and thermosensitive hydrogels can maintain a prolonged barrier within the submucosa, their barrier effects remain insufficient to meet the clinical requirement in some complicative endoscopic surgeries and their single structures pose challenges in implementing functional design. For example, some natural biogels can meet the needs of a physical barrier, but their relatively single structures limit their drug‐loaded and functional modification.^[^ ^6^, ^9^ ^]^ Hence, it is imperative to develop a drug‐loaded hydrogel with superior performance characteristics, such as easy injection due to shear‐thinning and strong shape retention due to in situ crosslinking, to ensure precise and safe endoscopic resection of gastrointestinal lesions and promote wound healing. However, injectability and shape stability are essentially difficult to balance. On the one hand, injectable hydrogels are usually diffcult to maintain a specific shape.^[^ ^10^ ^]^ On the other hand, the hydrogels in situ crosslinked to form a tough network have high in situ stability, but their operational complexity is a drawback.^[^ ^11^ ^]^ Additionally, the presence of residual cross‐linking agent and initiator during the cross‐linking reaction poses potential risks of tissue damage, thereby limiting their application in vivo.^[^ ^12^ ^]^ Therefore, the precise coupling of different or even opposite material properties and biological performances is highly desirable, yet remains a great challenge, to design high‐performance submucosal injection materials.

**Figure 1:**
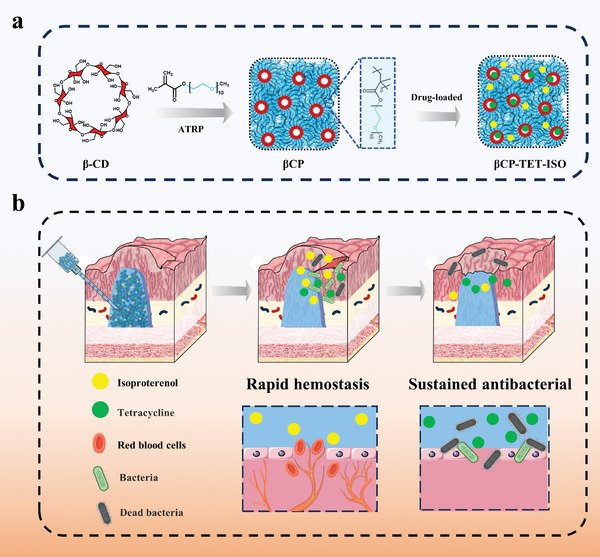
Schematic diagram of the multi‐arm star polymer hydrogel with shear‐thinning and drug loading capability. a) Preparation procedures and b) submucosal injection application of βCP‐TET‐ISO hydrogel. βCP‐TET‐ISO hydrogel exhibits the ability to establish a long‐lasting submucosal barrier for a long duration exceeding 120 min, which could satisfy the demands of intricate endoscopic procedures. This hydrogel can rapidly release ISO during the resection process to promote rapid hemostasis, and gradually release TET during the whole healing process to provide a continuous antibacterial effect and accelerate the healing process.

In this study, we have successfully developed a new class of multi‐arm star polymer hydrogel (denoted as βCP hydrogel) as an advanced submucosal injection material, which exhibits favorable properties such as good injectability, excellent in situ stability, and synergistic drug loading capability. As shown in **Figure** **1**a, βCP is synthesized by grafting poly(oligo(ethylene glycol) methacrylate) (PEGMA) side‐chains from β‐cyclodextrin (β‐CD) core via atom transfer radical polymerization (ATRP). During the onset of shearing, the entangled polymer brushes of βCP hydrogel undergo unwinding of polymer side‐chains between neighboring star brushes, resulting in a decrease in viscosity, which facilitates the process of endoscopic injectability. Once the injection shear is ceased after completing submucosal injection, the polymer brushes undergo winding of polymer side‐chains, thereby establishing a long‐lasting barrier cushion. Our βCP hydrogel can flow smoothly through a narrow and long endoscope needle (e.g., 25‐gauge, 1.8 m long), and entirely restore the robust hydrogel network once the injection shear is ceased, thereby providing a stable submucosal barrier for a long duration exceeding 120 min which can meet the requirements of intricate endoscopic procedures (typically lasting 60–120 min). In addition, owing to its distinctive configuration featuring a hydrophobic inner cavity of β‐CD and an outer layer of hydrophilic polymer side‐chains, βCP hydrogel enables simultaneous loading and on‐demand release of diverse categories of drugs, such as hydrophobic tetracycline (TET) and hydrophilic isoproterenol (ISO). According to Figure 1b, βCP hydrogel loaded with drugs (βCP‐TET‐ISO hydrogel) is able to promptly release the hydrophilic ISO during the resection, thereby facilitating rapid hemostasis of mucosal wound. Simultaneously, it gradually releases the hydrophobic TET during the healing process of mucosal wound, thereby providing an antibacterial effect and accelerating the healing process of mucosal wound. Our work could open a new avenue for the innovative design of superior materials for soft tissue barrier applications.

## 2 Results and Discussion

The star polymer βCP was synthesized by ATRP method. First, the β‐CD was esterified by 2‐bromoisobutyryl bromide to form an ATRP initiator β‐CD‐Br. The degree of conversion of the hydroxyl groups in β‐CD to the initiating sites in β‐CD‐Br is determined to be ≈85% from the ^1^H nuclear magnetic resonance (^1^H NMR) analysis (Figure S1a,b, Supporting Information), indicating that the number of initiating sites available for ATRP per molecule of β‐CD‐Br is ≈18. PEGMA side‐chains are grafted from the β‐CD‐Br via ATRP to form the βCP, which is confirmed by ^1^H NMR analysis (Figure S1c, Supporting Information). The number average molecular weight of PEGMA is 6.4 × 10^4^ Da (PDI = 1.31), which is determined by gel permeation chromatography (Figure S2, Supporting Information). In Fourier‐transform infrared (FTIR) spectrum (Figure S3, Supporting Information), the characteristic peaks of β‐CD are observed at 1035, 1080, and 1157 cm^−1^.^[^ ^13^ ^]^ After the esterification, the new characteristic peak at 1743 cm^−1^ appears in β‐CD‐Br, which is ascribed to C═O stretching. The symmetric stretching vibration peak of ‐CH_3_ in PEGMA is observed at 2865 cm^−1^, and the peak at 1725 cm^−1^ corresponds to the C═O absorption of the characteristic ester group.^[^ ^14^ ^]^ These findings provide evidence for the successful synthesis of βCP.

**Figure 2:**
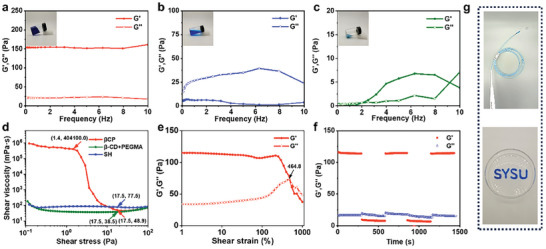
Rheology, shear‐thinning, and injectability properties of submucosal injected materials. Oscillation frequency scanning of a) βCP hydrogel, b) SH, and c) β‐CD+PEGMA. d) Shear viscosity‐stress curves of βCP hydrogel, SH, and β‐CD+PEGMA. e) Strain amplitude scanning and f) cyclic amplitude scanning of βCP hydrogel. g) Digital photos showing shear‐thinning injectability and good writing ability for βCP hydrogel with methylene blue staining.

The solid content of βCP hydrogels has a significant effect on their injection pressure. As shown in Figure S4 (Supporting Information), the injection pressure of βCP hydrogels increases with increasing their solid content. The injection pressure of βCP hydrogel with a solid content of 40 wt% is 51.9 Kpa (Figure S5, Supporting Information), which is the same level as that of the clinical SH solution (Figures S6, Supporting Information). Therefore, the βCP hydrogel with the solid content of 40 wt% is used for the following study. To investigate the shear‐thinning and self‐healing properties of βCP hydrogel, the rheological characterizations of βCP hydrogel and two control groups including SH and a mixture solution of β‐CD and PEGMA (denoted as β‐CD+PEGMA) were conducted. As shown in **Figure** **2**a, at a temperature of 25 °C, the energy storage modulus (G') of βCP hydrogel is consistently higher than its loss modulus (G'') across different frequencies, indicating that there is a stable gel network within βCP hydrogel.^[^ ^15^, ^16^, ^17^ ^]^ In contrast, for SH, G' is consistently lower than G'', suggesting that it exists in a solution state rather than a gel network (Figure 2b). As shown in Figure 2c, G' in β‐CD+PEGMA has the same order of magnitude as G'', suggesting a lack of a stable gel network. The shear viscosity curve depicted in Figure 2d exhibits the shear‐thinning properties of βCP hydrogel, as evidenced by the variations in viscosity under different shear forces. Specifically, as the shear force increases from 1.4 to 17.5 Pa, the viscosity of βCP hydrogel exhibits a rapid decrease from 404100.0 to 48.9 mPa·s (Figure 2d), thereby demonstrating its remarkable shear‐thinning characteristic. In the case of entangled βCP brushes, the initial reduction in viscosity during the onset of shearing can be attributed to the unwinding of polymer side‐chains between star polymers.^[^ ^18^ ^]^ In addition, βCP hydrogel also exhibits a remarkable self‐healing property. The strain amplitude scanning at a fixed frequency of 0.1 Hz at 25 °C reveals that the G' curve intersects the G'' curve when the shear strain reaches 464.8% (Figure 2e). Subsequently, with a continuous increase in shear strain, G' becomes smaller than G'', indicating the destruction of the gel network^[^ ^19^ ^]^ (Figure 2e). The cyclic amplitude scanning of βCP hydrogel in Figure 2f demonstrates that at low strain (1%), G' is greater than G'', indicating its gel state; at high strain (500%), G' decreases significantly and the gel network structure is destroyed (G' < G''). When the strain changes to low strain (1%) again, G' can quickly return to the initial state (Figure 2f).^[^ ^19^ ^]^ As shown in Figure 2g, βCP hydrogel can smoothly pass through an endoscopic injection needle with a length of 1.8 m and be easily injected into the desired shape.

**Figure 3:**
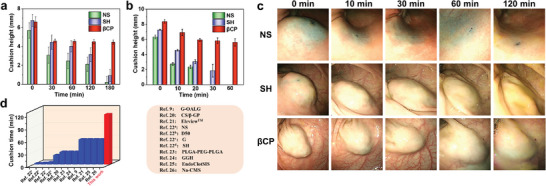
Shape retention ability of submucosal injected materials. Comparison of subcutaneous cushion height of NS, SH, and βCP hydrogel at different times after injection a) in the porcine stomachs in vitro and b) in rat models. c) Endoscopic photos of the submucosal cushions with NS, SH, and βCP hydrogel at 30, 60, and 120 min after injection in the porcine stomach in vivo. d) Comparison of βCP hydrogel with the reported submucosal injected materials in terms of cushion time in porcine stomachs in vivo.^[^ ^9^, ^20^, ^21^, ^22^, ^23^, ^24^, ^25^, ^26^ ^]^

In addition to the easy injection performance, our injected βCP hydrogel also demonstrates a strong shape retention capacity, because the hydrogel could entirely restore the gel state and stably remain in place after shear force is absent (Figure S7, Supporting Information). Microscopic observations for the samples treated by freeze‐drying were implemented by scanning electron microscopy (SEM). Our βCP hydrogel has homogeneous and smooth surface morphology (Figure S8a, Supporting Information); on the contrary, the submucosal tissue has loosely porous and rough structure (Figure S8b, Supporting Information). The cross‐sectional SEM image further confirms that βCP hydrogel and submucosal tissue have significantly different pore structures, and are tightly connected with each other to form a seamless interface (Figure S8c, Supporting Information), which is attributed to the slight infiltration of βCP hydrogel into porous structure of submucosal tissue before the in situ cross‐linking. As shown in **Figure** **3**a, after the injection of βCP hydrogel, NS, and SH into the submucosal tissue of in vitro porcine stomach, the cushion height in different groups is recorded at 0, 30, 60, 120, and 180 min. Within 30 min after injection, the cushion height of βCP hydrogel group shows only a slight decrease, and with further extending the duration to 180 min, the cushion height still keeps 67.5% of its initial value (Figure 3a). In sharp contrast, the cushion height of the NS and SH groups rapidly decreases over time, with significant reductions to only 3.5% and 13.6% of the initial height, respectively, after 180 min of injection (Figure S9, Supporting Information). Subsequently, βCP hydrogel, NS, and SH were injected subcutaneously on the dorsal region of rats, and the degrees of elevation were assessed at 10, 20, 30, and 60 min after injection. Consistent with the results of the in vitro experiment using porcine stomach, the cushion height of βCP hydrogel exhibits a minimal alteration at 20 min after injection and still keeps 66.9% of its initial height at 60 min after injection (Figure 3b). Conversely, the cushion heights of the NS and SH groups show a significant decrease within 20 min and are unquantifiable at 60 min after injection (Figure 3b). To further examine the submucosal barrier properties of different substances in large animal, we conducted an endoscopy experiment involving the in vivo submucosal injection of porcine stomach. After the injection of 3.0 mL of βCP hydrogel into the gastric submucosa using an endoscopy device, the cushion height of βCP hydrogel exhibits a minimal change within 30 min, and an obvious barrier remains visible even after 120 min (Figure 3c). In contrast, the NS group demonstrates a notable decline in cushion height within 10 min after injection, and its height is difficult to observe at 30 min after injection. The cushion height of SH group shows a relative slow downward trend, but only a slight cushion could be observed at 120 min (Figure 3c). What's more, as shown in Figure 3d, βCP hydrogel displays aa significantly superior barrier effect in comparison to that of the reported submucosal injection materials (such as 30 min for SH^[^ ^22^ ^]^ and GGH,^[^ ^24^ ^]^ as well as 60 min for EndoClotSIS^[^ ^25^ ^]^).

**Figure 4:**
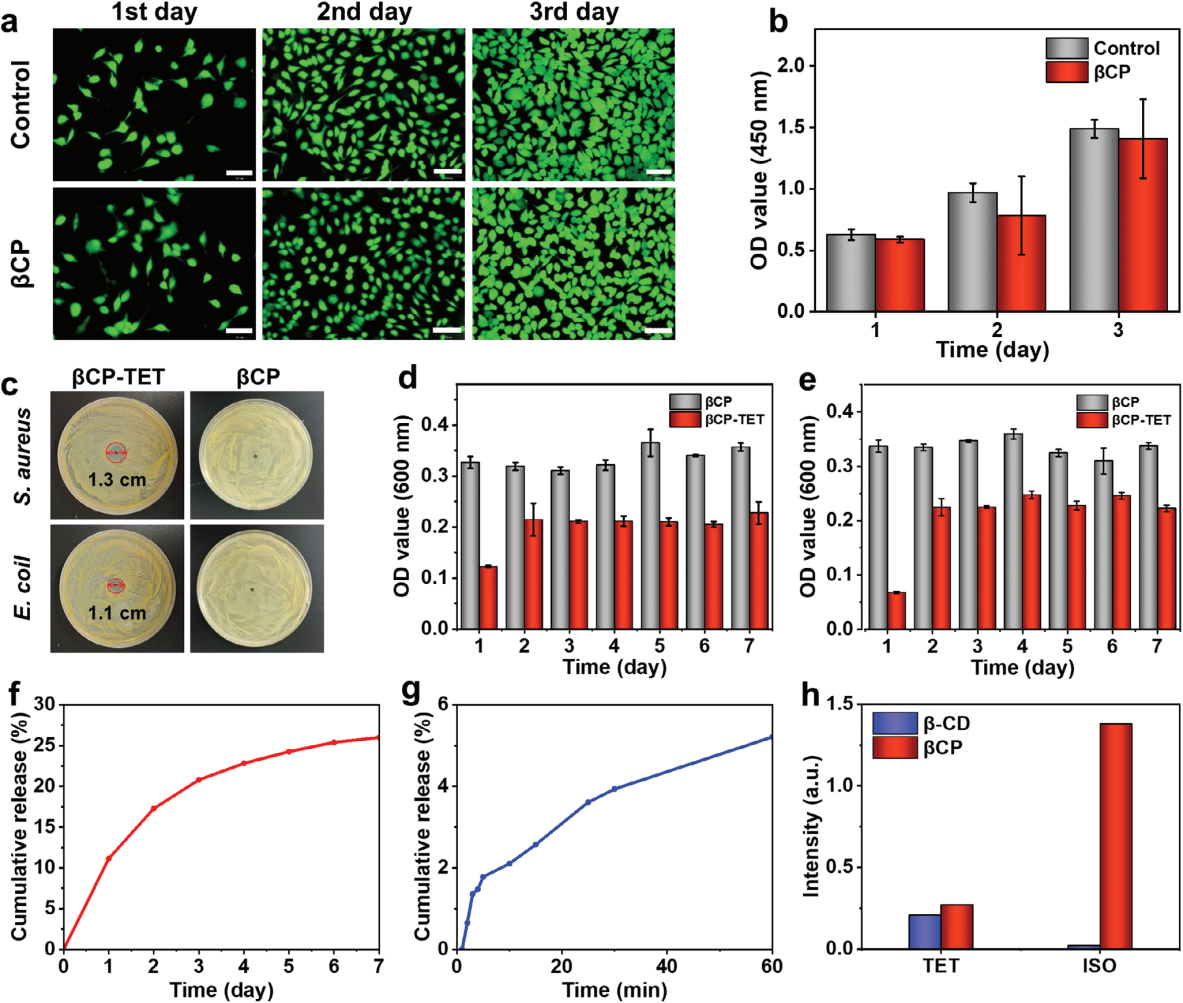
a) In vitro fluorescence images and b) CCK‐8 assay of L929 fibroblasts co‐cultured after 1, 2, and 3 days with the control and βCP hydrogel groups. Scale bars: 100 µm. c) Digital photos of inhibition zones of βCP‐TET hydrogel for *E. coli* and *S. aureus* at 24 h. The sustained antibacterial activity of βCP‐TET hydrogel against d) *E. coli* and e) *S. aureus* within 7 days. Cumulative release curves of f) TET from βCP‐TET hydrogel and g) ISO from βCP‐ISO hydrogel. h) Cumulative release of TET or ISO from β‐CD and βCP hydrogel within 24 h.

Live/dead cell double staining kits and Cell Counting Kit‐8 (CCK‐8) were used to evaluate the biocompatibility of the materials. According to the live/dead staining images, L929 fibroblasts in the βCP hydrogel group exhibit similar morphology and cell density to the control group (**Figure** **4**a). Similarly, L929 fibroblasts in the βCP‐TET‐ISO hydrogel group also exhibit similar morphology and cell density to the control group (Figure S10a, Supporting Information). In addition, the quantitative analysis of the CCK‐8 assay of the βCP and βCP‐TET‐ISO groups shows no significant difference compared with the control group (Figure 4b and Figure S10b, Supporting Information), demonstrating the good biocompatibility of βCP‐TET‐ISO hydrogel. To further illustrate the biocompatibility in vivo, βCP hydrogel was injected subcutaneously on the dorsal region of rats to form a cutaneous hillock ≈2.0 cm in diameter. The subcutaneous tissue containing βCP hydrogel was cut into slices and assessed by hematoxylin‐eosin (H&E) staining. Local infiltration of inflammatory cells is found inside and around the hydrogel on day 1; however, the inflammatory response attenuates to slight level on day 3, and a very few inflammatory cells can be found on day 7 (Figure S11, Supporting Information). The result confirms the acceptable in vivo biocompatibility of βCP hydrogel. During the endoscopic resection of digestive tract lesions, complications such as bleeding and infection are prone to occur, thereby leading to poor wound healing.^[^ ^27^, ^28^ ^]^ It is worth noting that ISO is a commonly used hydrophilic hemostatic drug, while TET is a hydrophobic antibacterial drug. Therefore, the simultaneous loading and controlled release of these hydrophobic and hydrophilic drugs in submucosal injection materials pose a significant challenge. To facilitate the process of wound healing, we further explored the drug loading properties of submucosal injection materials. The antibacterial properties of the hydrophobic drug TET‐loaded βCP hydrogel (denoted as βCP‐TET hydrogel) were assessed using the antibacterial ring method. Following a 24 h co‐culture period, βCP‐TET hydrogel exhibits inhibitory effects against both *Escherichia coli* (*E. coli*) and *Staphylococcus aureus* (*S. aureus*), resulting in the formation of inhibitory rings with 1.1 and 1.3 cm in diameter, respectively, while no inhibition zone is observed in βCP hydrogel (Figure 4c). To provide a more comprehensive understanding of the material's antibacterial performance, we conducted a co‐culture experiment by incubating different samples (βCP‐TET and βCP hydrogels) with *E. coli* or *S. aureus* for a duration of 7 days. The bacterial solution was replaced with a fresh one every 24 h, and the optical density (OD) value of the bacterial solution was measured and recorded. The OD value of the βCP‐TET hydrogel group is consistently lower than that of the βCP hydrogel group without TET within 7 days. This result suggests that βCP‐TET hydrogel exhibits a sustained bacteriostatic effect on both *E. coli* or *S. aureus* (Figure 4d,e). In order to assess the release kinetics of TET, we conducted the drug release measurements. Our βCP‐TET hydrogel exhibits a gradual release pattern, with a release of only 11.1% on the first day, followed by a release of 26.0% after 7 days (Figure 4f), indicating its slow‐release property for TET. We further evaluated the drug release behavior of the hydrophilic drug ISO‐loaded βCP hydrogel (denoted as βCP‐ISO hydrogel). In contrast to a gradual release profile observed in βCP‐TET hydrogel, βCP‐ISO hydrogel demonstrates a significantly rapid release of 5.2% within 60 min for ISO (Figure 4g). Furthermore, the drug cumulative release curve of βCP‐ISO hydrogel demonstrates a release of 41.3% on the first day and 68.3% after 7 days (Figure S12, Supporting Information). In order to investigate the different release behaviors of hydrophobic and hydrophilic drugs, a comparative analysis was conducted between the drug release profiles of βCP hydrogel and β‐CD (a control sample without grafted side‐chains). As shown in Figure 4h, β‐CD with a hydrophobic cavity shows a significantly lower release amount of the hydrophilic drug ISO, accounting for only 1.6% of the release amount of βCP hydrogel after 24 h. Conversely, β‐CD demonstrates a favorable drug release performance for the hydrophobic drug TET, accounting for as high as 75.6% of the release amount of βCP hydrogel after 24 h (Figure 4h). The effective TET payload for β‐CD can be attributed to the distinctive hydrophobic central cavity of β‐CD, which allows for the partial or complete inclusion of hydrophobic drugs within its hydrophobic central cavity, thereby achieving the effective loading and slow release of hydrophobic drugs.^[^ ^29^, ^30^ ^]^ Meanwhile, the hydrophilic drug ISO is more prone to interacting with the hydrophilic side‐chains of βCP hydrogel, facilitating high drug loading and releasing.^[^ ^31^ ^]^ Therefore, the unique structural design of our βCP hydrogel holds promising potential for effectively accomplishing the simultaneous loading and controlled release of both hydrophilic and hydrophobic drugs, and is expected to meet the diverse requirements of gastrointestinal wound healing during different stages.

**Figure 5:**
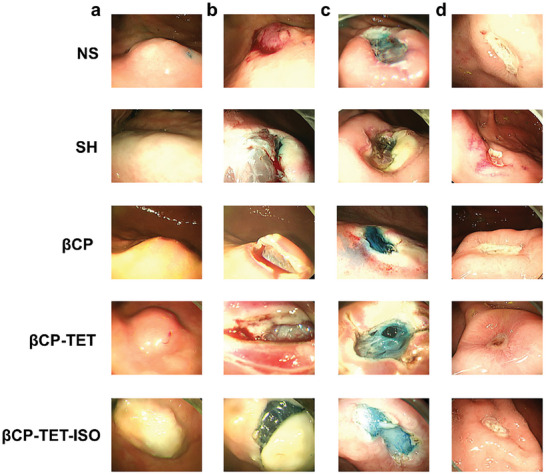
Endoscopic photos of a) submucosal cushions after the endoscopic injection, b) hemostatic status during the mucosal resection by the endoscope electrosurgical system, c) mucosal wounds right after the resection, and d) mucosal wounds after the resection for 7 days with NS, SH, βCP hydrogel, βCP‐TET hydrogel, and βCP‐TET‐ISO hydrogel.

To validate the physical barrier properties of βCP hydrogel in the resection of gastrointestinal lesions and its potential in promoting wound healing, we conducted an in vivo gastric endoscopic resection on a porcine model. Firstly, we obtained a βCP‐TET‐ISO hydrogel by incorporating TET and ISO drugs into βCP hydrogel. 2.0 mL of methylene blue‐stained NS, SH, βCP hydrogel, βCP‐TET hydrogel or βCP‐TET‐ISO hydrogel was injected into the submucosa of the porcine stomach via a gastroscope (**Figure** **5**a). Subsequently, the elevated mucosal tissue was excised using an endoscopic surgical system. As shown in Figure 5b,c, βCP‐TET‐ISO hydrogel exhibits a remarkable hemostatic effect during resection owing to its rapid release of ISO. However, wound bleeding is observed in the NS, SH, βCP hydrogel, and βCP‐TET hydrogel groups during the resection of mucosal tissue (Figure 5b). To further assess the impact of βCP‐TET‐ISO hydrogel on the wound healing process, a subsequent endoscopy was conducted on the 7th day after the endoscopic resection. The βCP‐TET hydrogel and βCP‐TET‐ISO hydrogel groups exhibit enhanced wound healing outcomes in comparison to the NS, SH, and βCP hydrogel groups, as illustrated in Figure 5d. This could be attributed to the gradual release of TET, leading to a reduction of inflammatory response in the gastric lesions. These results highlight the rapid hemostatic properties of βCP‐TET‐ISO hydrogel, as well as its ability to facilitate wound healing. It is worth mentioning that addition of TET and ISO has no adverse effect on the gel network and shear‐thinning characteristics of βCP hydrogel; there is no significant difference in comparison of G′ and G″ for βCP and βCP‐TET‐ISO hydrogels. For example, their shear viscosity‐stress curve almost coincide; like βCP hydrogel, βCP‐TET‐ISO hydrogel can quickly undergo sol‐gel transition after injection with endoscopic needle (Figure S13, Supporting Information).

## 3 Conclusion

In summary, we report the development and application of βCP hydrogel with good injectability, excellent in situ stability, and synergistic drug loading properties. βCP can be successfully synthesized as the submucosal injection material by grafting PEGMA from β‐CD core via ATRP. Benefiting from the unique multi‐arm star structure, our βCP hydrogel shows good shear‐thinning property, and simultaneously has superior submucosal barrier performance for a long duration exceeding 120 min and versatile drug loading and on‐demand release capabilities. Remarkably, owing to its distinctive configuration featuring an outer layer of hydrophilic polymer side‐chains and a hydrophobic inner cavity of β‐CD, βCP hydrogel exhibits the hydrophilic ISO release of 37.4% after 24 h and the hydrophobic TET release of 33.7% after 7 days. The differential release rates can effectively fulfill the hemostatic requirement during the procedure of resection and the antibacterial demand during the whole healing process. Based on these characteristics, our βCP hydrogel could be a promising material for broad applications in endoscopic resection techniques, as well as potential development in drug delivery and tissue engineering.

## Conflict of Interest

The authors declare no conflict of interest.

## Supporting information

Supporting Information

## Data Availability

The data that support the findings of this study are available from the corresponding author upon reasonable request.
