# Supplementary material for: Synergistic Drug‐Loaded Shear‐Thinning Star Polymer Hydrogel Facilitates Gastrointestinal Lesion Resection and Promotes Wound Healing
Source: Adv Sci (Weinh). 2024 Apr 30;11(26):2309586. doi: 10.1002/advs.202309586 (PMC11234439; doi:10.1002/advs.202309586)
Supplement: Supplementary file 1 — Supporting Information [file ADVS-11-2309586-s001.pdf]

ADVANCED SCIENCE
Open Access

Supporting Information

for *Adv. Sci.*, DOI 10.1002/advs.202309586

Synergistic Drug-Loaded Shear-Thinning Star Polymer Hydrogel Facilitates Gastrointestinal Lesion Resection and Promotes Wound Healing

Yue Zhang, Dongtian Miao, Mingli Su, Yinxiang Tang, Minghong Zhou, Yang Yu, Xuefeng Guo* and Dingcai Wu**

Supporting Information

Synergistic Drug-Loaded Shear-Thinning Star Polymer Hydrogel Facilitates Gastrointestinal Lesion Resection and Promotes Wound Healing

Yue Zhang, Dongtian Miao, Mingli Su, Yinxiang Tang, Minghong Zhou, Yang Yu, Xuefeng Guo*, Dingcai Wu**

Y. Zhang, D. Miao, Y. Tang, Prof. D. Wu

PCFM Lab, School of Chemistry, Sun Yat-sen University, Guangzhou 510006, P. R. China

E-mail: wudc@mail.sysu.edu.cn

M. Su, Dr. X. Guo

Department of General Surgery (Endoscopic Surgery), Guangdong Provincial Key Laboratory of Colorectal and Pelvic Floor Diseases, Biomedical Innovation Center, Key Laboratory of Human Microbiome and Chronic Diseases (Sun Yat-sen University), Ministry of Education, Guangzhou 510655, P. R. China

The Sixth Affiliated Hospital, Sun Yat-sen University, Guangzhou 510655, P. R. China

E-mail: guoxfeng@mail.sysu.edu.cn

Dr. M. Zhou

Medical Research Institute, Guangdong Provincial People's Hospital (Guangdong Academy of Medical Sciences), Southern Medical University, Guangzhou 510080, P. R. China

E-mail: zhouminghong@gdph.org.cn

Y. Yu

Department of General Surgery (Colorectal Surgery), Guangdong Institute of Gastroenterology, Biomedical Innovation Center, Guangdong Provincial Key Laboratory of Colorectal and Pelvic Floor Diseases, The Sixth Affiliated Hospital, Sun Yat-sen University, Guangzhou 510655, P. R. China

Materials

β-Cyclodextrin (β-CD) was obtained from Sigma-Aldrich. Oligo(ethylene glycol) methyl ether methacrylate (OEGMA), tin(II) 2-ethylhexanoate, and 4-(dimethylamino)-pyridine (DMAP) were purchased from Shanghai Aladdin Biochemical Technology Co., Ltd. 2-Bromoisobutyryl bromide (BiBB), *N,N,N',N",N"*-pentamethyldiethylenetriamine (PMDETA), and ethyl 2-bromoisobutyrate (EBiB) were purchased from J&K Scientific Ltd. Anhydrous triethylamine, sodium bicarbonate ($NaHCO_3$), magnesium sulfate anhydrous ($MgSO_4$), *N,N*-dimethylformamide, tetracycline (TET), hexane, and anhydrous chloroform were purchased from Shanghai Macklin Biochemical Co., Ltd. Phosphate buffer saline (PBS, 0.01 M, pH 7.4) was purchased from Dalian Meilun Biotech Co., Ltd. Dialysis bag (3500 Da) was obtained from Shanghai Yuanye Bio-Technology Co., Ltd. Glycerol fructose sodium chloride injection (GF) was purchased from Jiangsu Chia Tai Fenghai Pharmaceutical Co., Ltd. Isoproterenol (ISO, 1 mg mL^{-1}) was purchased from Grand Pharma (China) Co., Ltd. Methylene blue injection (10 mg mL^{-1}) was purchased from Jiangsu Jichuan Pharmaceutical Co., Ltd. Endoscopic needle (VDK-IN-23-180-2504-A) was purchased form Jiangsu Vedkang Medical Science and Technology Co., Ltd. The Cell Counting Kit-8 (CCK-8) and Calcein-AM/PI Double Staining Kits were purchased from Beijing Solarbio Science & Technology Co., Ltd. L929 fibroblasts was provided by the Procell Life Science & Technology Co., Ltd. *Staphylococcus aureus* (*S. aureus*, ATCC 25923) and *Escherichia coli* (*E. coli*, ATCC 25922) were obtained from Guangdong Provincial People's Hospital. OEGMA was purified by passage through a basic alumina column, β-CD was dried in a vacuum oven at 80 ºC for 2 h before use, and others were used as received.

Synthesis of β-CD-Br

Dried β-CD (1135 mg) was added into Schlenk flask equipped with a rubber septum and purged with N_2. Subsequently, 4 mL of anhydrous chloroform containing DMAP (150 mg) and anhydrous triethylamine (8.3 mL) were injected into the flask and kept

under stirring for 1 h. Next, 6.2 mL of BiBB was added dropwise for 30 min under an ice bath. The mixture was placed to room temperature and the reaction was kept under stirring for 24 h. The reacted mixture was filtered and washed successively with deionized (DI) water, $NaHCO_3$ saturated solution, and DI water. Purified organic phase was dried with $MgSO_4$, and the solvent was removed under reduced pressure. The crude product was redissolved in chloroform and precipitated in hexane. The obtained β-CD-Br was dried in a vacuum oven at 40 ºC for 24 h.

Synthesis of βCP, βCP-TET, βCP-ISO, and βCP-TET-ISO hydrogels

50 mg of β-CD-Br was placed in a 25 mL Schlenk flask containing 10 mg of $CuBr_2$, 100 μL of PMDETA, 5 mL of OEGMA, and 3 mL of *N,N*-dimethylformamide. N_2 was bubbled for 30 min and 1.0 mL of *N,N*-dimethylformamide containing 100 μL of tin(II) 2-ethylhexanoate was injected into the flask. The flask was immersed in an oil bath at 60 ºC for 30 min, and was then removed from the oil bath and exposed to air to stop the reaction. Thereafter, the reaction solution was placed in dialysis bag (3500 Da) and dialyzed in 2000 mL of DI water for 3 days. The obtained solution was freeze-dried to give the star polymer β-CD-*g*-PEGMA (βCP). βCP hydrogels were prepared by combining DI water with freeze-dried βCP in different weight ratios.

βCP-TET hydrogel was prepared by mixing TET solution (0.1 mg mL^{-1}) with freeze-dried βCP in a weight ratio of 6:4, followed by shaking at 37 °C with 120 rpm for a duration of 12 h. βCP-ISO and βCP-TET-ISO hydrogels were obtained via the same procedures as βCP-TET hydrogel, except for replacing the TET solution with the ISO solution (0.01 mg mL^{-1}) and the mixture solution (0.1 mg mL^{-1} TET and 0.01 mg mL^{-1} ISO), respectively.

Preparation of linear PEGMA and β-CD+PEGMA

46 mg of EBiB was placed in a 25 mL Schlenk flask containing 10 mg of $CuBr_2$, 100 μL of PMDETA, 5 mL of OEGMA, and 3 mL of *N,N*-dimethylformamide. N_2 was bubbled for 30 min and 1.0 mL of *N,N*-dimethylformamide containing 100 μL of tin(II) 2-ethylhexanoate was injected into the flask. The flask was immersed in an oil bath at 60 ºC for 30 min, and was then precipitated in ether. The obtained precipitation

was dried in a vacuum oven at 40 ºC for 12 h, producing the linear PEGMA. The β-CD+PEGMA was obtained by mixing 5 mg of β-CD, 5 g of linear PEGMA, and 7.5 g of DI water.

Material characterization

Microstructures were investigated by scanning electron microscopy (SEM, S-4800, Hitachi). Before SEM observation, the samples were freeze-dried and sprayed with platinum. Fourier-transform infrared (FTIR) spectra were recorded on FTIR spectrometer (Tensor 27, Bruker). Proton nuclear magnetic resonance (^{1}H NMR) spectra were performed on Nuclear Magnetic Resonance 400 M (Bruker advance III, Bruker BioSpin). The molecular weight was analyzed by gel permeation chromatography (GPC, Breeze GPC, Waters). DI water was used as a solvent for polymers and eluent for GPC with a flow speed of 0.6 mL min^{-1} at 35 ºC.

Measurement of rheological and viscosity properties for βCP hydrogel

Shear viscosity, shear strain, energy storage modulus, and loss modulus were obtained using a rotational rheometer (Kinexus pro+, Malvern). According to the guidelines of instrument, βCP and βCP-TET-ISO hydrogels were put on a rotating disk. All rheological tests were performed at 25 °C. Oscillation frequency scanning was conducted at a strain of 1.0% and frequency range of 0.1-10.0 Hz. Shear viscosity curve was obtained through a yield viscosity measurement at a strain of 1.0% and fixed frequency of 0.1 Hz. Shear stress range and action time were set as 0.1-100.0 Pa and 10 min, respectively. Strain amplitude scanning (0.1-1000%) was conducted at a fixed frequency of 0.1 Hz. The cyclic amplitude scanning was performed as follows: First, βCP hydrogel was tested with a strain of 1%. Subsequently, the strain was changed from 1% to 500% and remained at 500% for 5 min to completely damage the gel networks. Finally, the strain was reduced from 500% to 1% and remained at 1% for 5 min to observe whether the gel networks were restored.

Drug-release profile in vitro

In order to investigate the potential application of βCP hydrogel as drug carriers, hydrophobic TET and hydrophilic ISO were utilized for in vitro release studies. The

in vitro release amounts of TET from βCP-TET hydrogel and ISO from βCP-ISO hydrogel were determined using a dialysis method. 1 mL of βCP-TET and βCP-ISO hydrogels were placed into dialysis bags and incubated in 10 mL DI water at 37 °C with 120 rpm shaking, respectively. At different time points, all release solutions were harvested and replaced with same volumes of DI water. The release amounts of TET and ISO were calculated by absorbance at 350 nm and 280 nm, respectively, using ultraviolet-visible absorption spectra (Lambda 950, PerkinElmer).

Measurement of injection pressure of βCP hydrogel

Injection pressure of NS, GF, SH, and βCP hydrogel were evaluated with a measurement system, in which an injection needle (18-gauge), a digital pressure gauge (HT-1895, Xinsite) and a syringe were connected to a three-way stopcock, followed by fixing the syringe with a syringe pump (LSP01-1A, Longer) to set injection speed. Injection pressure was measured by a 1 mL syringe at an injection speed of 1 mL min^{-1}. Six independent injection pressure measurements were performed for each sample.

Biocompatibility assessments of βCP hydrogel in vitro

Proliferation of L929 fibroblast cells were assessed by CCK-8 method. The samples were sterilized using UV irradiation for 1 h and then immersed in complete medium for 24 h to obtain hydrogel extracts with the concentrations of 200 mg mL^{-1}. Briefly, the experiment was divided into the control, βCP hydrogel, and βCP-TET-ISO hydrogel groups. In the control group, L929 fibroblast cells were seeded into 96-well plates with a density of 5000 cells in 100 μL per well and incubated for 1, 2, and 3 days at 37 °C in the 5% CO_2 humidified incubator (Forma Steri-cycle, Thermo) to obtain a monolayer of cells. For the βCP hydrogel and βCP-TET-ISO hydrogel groups, 100 μL of βCP hydrogel or βCP-TET-ISO extracts was added into 96-well plate after seeding cells, and the cells were then incubated for 1, 2, and 3 days under the same conditions as the control group. After incubation, culture medium was removed, and 100 μL of fresh medium (10 μL of CCK-8 reagent) was added into each well and then incubated at 37 °C for 2 h. Thereafter, optical density (OD) values of samples were

measured by a microplate reader (Multiskan FC, Thermo) at 450 nm.

For live/dead cell double staining kits experiment, the groups were divided as above. For the control group, L929 fibroblast cells were seeded into 12-well plates with a density of 5000 cells in 1 mL per well and incubated for 1, 2, and 3 days at 37 °C in the 5% CO_2 humidified incubator. For the βCP hydrogel and βCP-TET-ISO hydrogel groups, 100 μL of βCP hydrogel or βCP-TET-ISO hydrogel extracts was added into the plates after seeding cells, respectively, and then the cells were incubated for 1, 2, and 3 days. After incubation, culture medium was removed and every well was washed with PBS for three times, and then 100 μL of Calcein-AM (2 μg mL^{-1}) solution was added into each well and incubated for 30 min. Thereafter, each well was washed by PBS and observed by an inversed fluorescent microscope (IX73, Olympus).

Antibacterial properties of βCP-TET hydrogel

For the antibacterial test, *S. aureus* and *E. coli* were precultured in sterilized Luria-Bertani broth (LB) at 37 °C for 12 h to obtain a concentration of approximately 10^7 CFU mL^{-1}. To determine the zone of inhibition, 100 μL of bacterial suspension was spread uniformly over the agar plates. βCP and βCP-TET hydrogels (diameter 1 mm) were put into every LB agar plate. The inoculated agars were incubated at 37 °C. The diameter of the inhibition zone was recorded at 24 h after incubation. For the co-culture experiment, 0.25 mL βCP and βCP-TET hydrogels were placed in dialysis bags (3500 Da), respectively. The dialysis bags were incubated in 5 mL of bacterial suspension with a concentration of 10^5 CFU mL^{-1} and then cultured at 37 °C with 120 rpm shaking. The dialysis bags were taken out and washed three times with DI water and then added to new 5 mL bacterial suspension every 24 h. After that, the OD values of the former residual bacterial suspensions were measured every 24 h.

Measurement of submucosal cushion height in porcine stomach in vitro

Submucosal cushion height was measured with the digital display micrometer thickness gauge. Gastric specimens were collected from the upper third of the fresh porcine stomachs, whose thickness was close to that of the human stomach. 1.0 mL of

βCP hydrogel, NS or SH was injected into submucosa from center of specimen by using a 1 mL syringe with a 26-gauge needle. Cushion heights were measured at 0, 10, 30, 60, 120, and 180 min after injection. Five independent measurements were performed, and the obtained results were expressed as the mean ± standard deviation. Height retention rate of submucosal cushion at different times after injection was calculated, according to the following equation:

$$\textit{Height retention rate}\ (\%) = \frac{\textit{Mean cushion height at measurement time}}{\textit{Mean cushion height at initial time}} \times 100\%$$

Measurement of submucosal cushion height in rat model

Sprague-Dawley rats (200 ~ 240 g, Sun Yat-sen University) were used for all in vivo studies of rats. All the experimental operations involved in rats were approved by the guidelines of the Animal Ethics Committee for Guangzhou Huateng Biomedical Technology Co., Ltd. (license number: H7SW220828). Anesthesia was induced with intraperitoneal injection of abdominal anesthesia (3% sodium pentobarbital, 1.5 mL kg^{-1}). Firstly, dorsal region of rat was prepared for subcutaneous injection by clipping the hair and cleaning the operative area with medicinal alcohol solution. Subsequently, 1.0 mL of NS, SH, or βCP hydrogel was injected into the dorsal skin under sterile condition. After 1, 3, and 7 days, the rats were sacrificed by abdominal anesthesia. Subcutaneous regions of interest were excised and fixed in 4% paraformaldehyde solution for 24 h, and then sent to Wuhan Servicebio Technology Co., Ltd. for hematoxylin-eosin (H&E) staining. After placing a ruler next to cushion, the cushion height was measured with the ruler at 10, 20, 30, and 60 min after injection. Four rats in each group were tested in parallel.

Performance of βCP hydrogel in porcine model

A Tibet miniature pig (30 ~ 40 kg) was provided by Guangzhou Liming Bio-Technology Co., Ltd. All the experimental procedures involved in pig were approved by the guidelines of the Animal Ethics Committee for Yin She Guangzhou Medical Technology Co., Ltd. (license number: SS-2022-ZSL 1). The pig was treated following the Laboratory Animal Care and Use Guidelines strictly. The pig was only allowed to drink water at 2 days before experiment. The pig was anesthetized by

xylazine hydrochloride and propofol, and endotracheal intubation was performed with continuous oxygen intake of 2 L min^{-1}. An endoscope was entered into the porcine stomach for observation. Subsequently, 3.0 mL of NS, SH, or βCP hydrogel was injected into submucosa with an endoscopic needle (VDK-IN-23-180-2504-A, Vedkang) at front wall or back wall of the gastric antrum. Thereafter, shape change of submucosal cushion was observed by the endoscope at 0, 10, 30, 60, and 120 min after endoscopic injection, and the obtained digital photos were recorded and analyzed. On the other hand, 2.0 mL of NS, SH, βCP hydrogel, or βCP-TET-ISO hydrogel was injected into submucosa and then mucosae were resected by an electrosurgical knife (KD-655L, Olympus). The obtained digital photos were recorded to observe the hemostatic effect. The evaluation of wound healing progress was conducted using an endoscope at 7 days after resection.

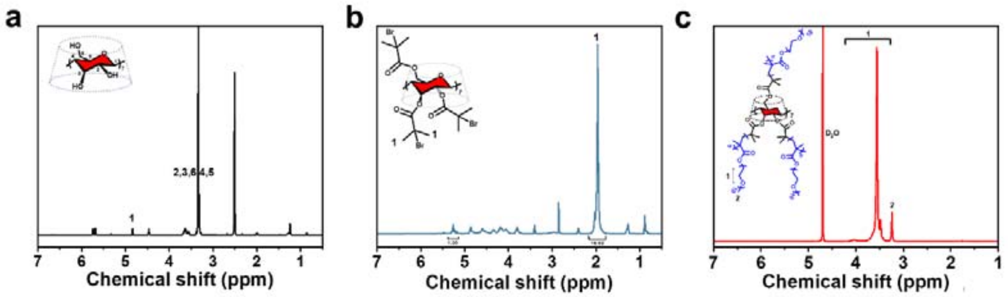

Figure S1. ^{1}H NMR spectra of a) β-CD in DMSO-d6, b) β-CD-Br in $CDCl_3$, and c) βCP in D_2O.

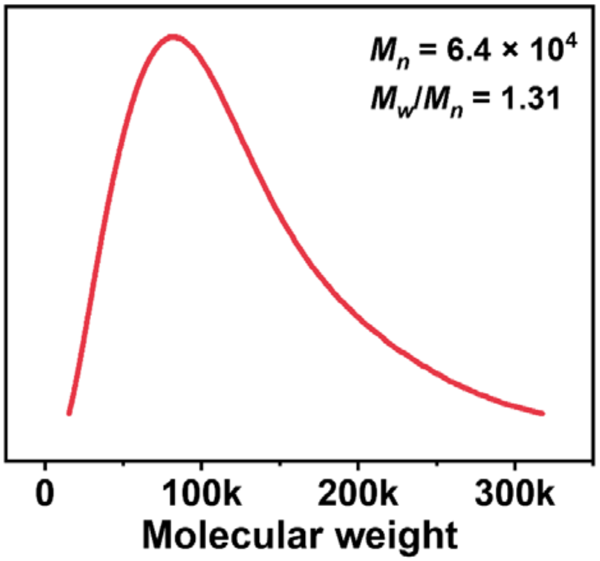

Figure S2. GPC trace of linear PEGMA.

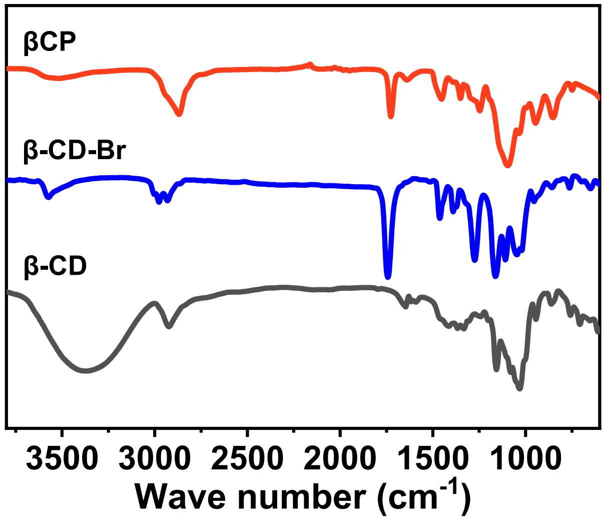

Figure S3. FTIR spectra of β-CD, β-CD-Br, and βCP.

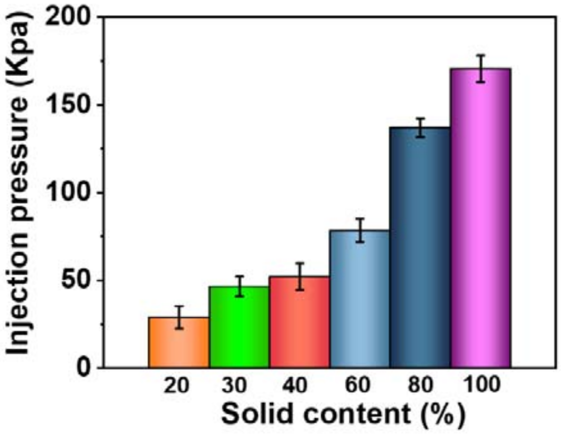

Figure S4. Injection pressure of βCP hydrogels with different solid contents.

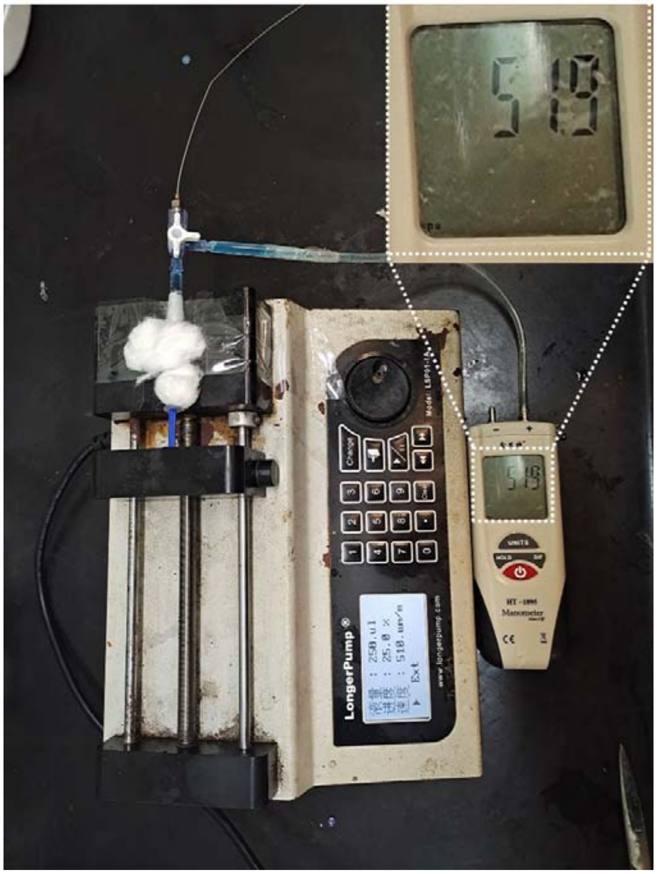

Figure S5. Injection pressure of βCP hydrogel with 40 wt% solid content.

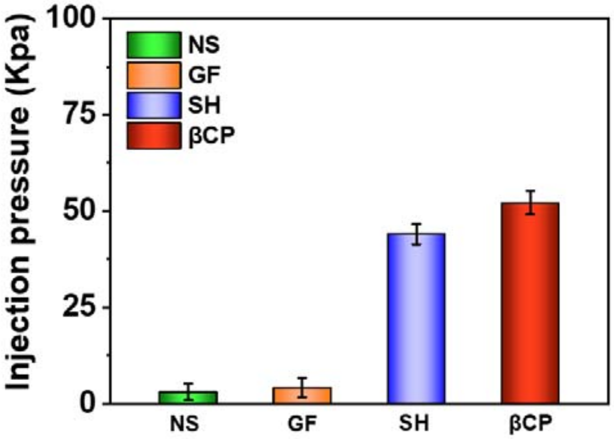

Figure S6. Comparation of injection pressure of NS, GF, SH, and βCP hydrogel.

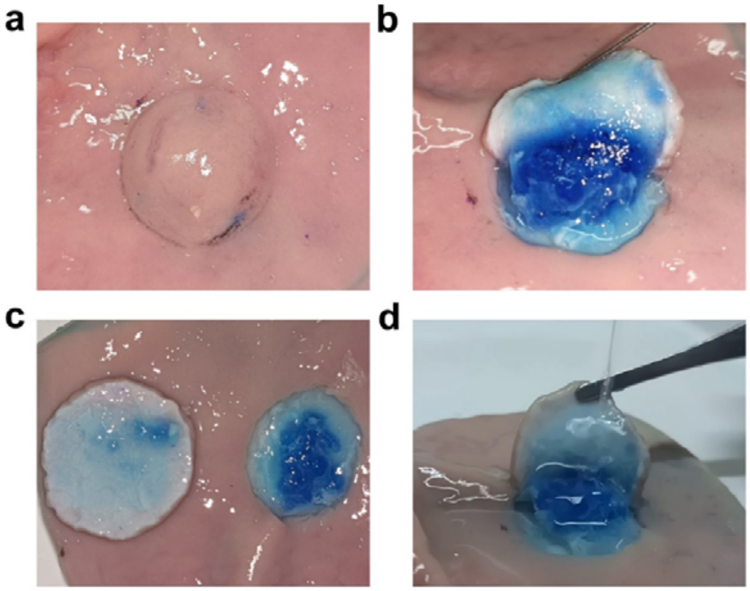

Figure S7. Digital photos of a) submucosal cushion after injection of βCP hydrogel, b) βCP hydrogel between the mucosa layer and intrinsic muscle layer during mucosal resection, c) βCP hydrogel on the intrinsic muscle layer after resection, and d) βCP hydrogel that is being washed with flowing water during mucosal resection.

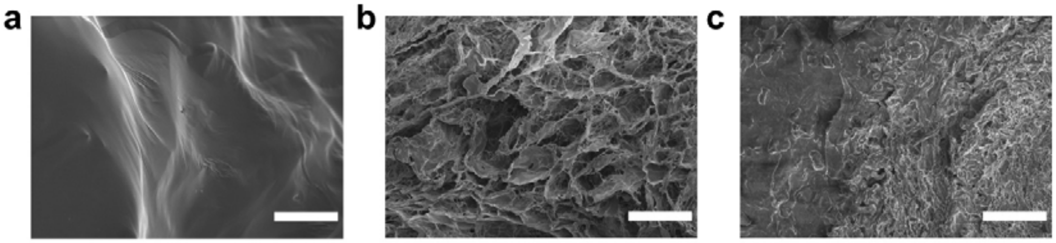

Figure S8. SEM images of a) freeze-dried βCP hydrogel, b) freeze-dried submucosal tissue, and c) the interface between freeze-dried βCP hydrogel and submucosal tissue. Scale bars: 50 μm.

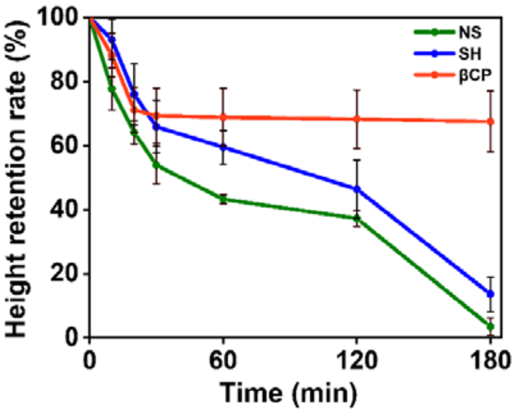

Figure S9. Height retention rates of NS, SH, and βCP hydrogel at different times.

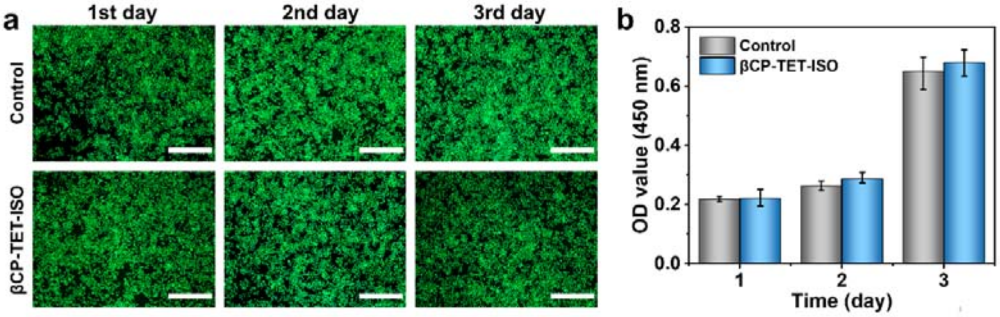

Figure S10. a) In vitro fluorescence images and b) CCK-8 assay of L929 fibroblasts co-cultured after 1, 2, and 3 days with the control and βCP-TET-ISO hydrogel groups. Scale bars: 500 μm.

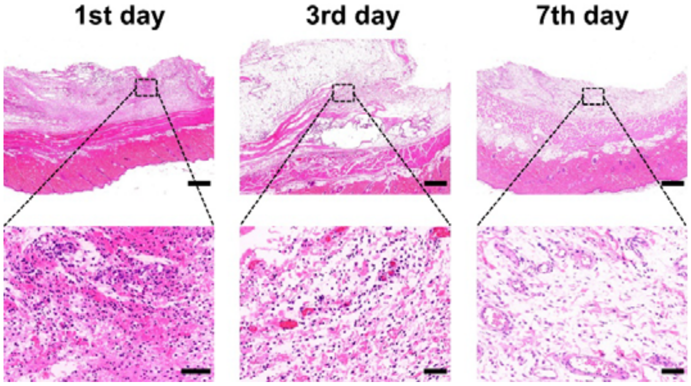

Figure S11. H&E staining of βCP hydrogel group at different times. Upper bars: 2 mm, bottom bars: 200 μm.

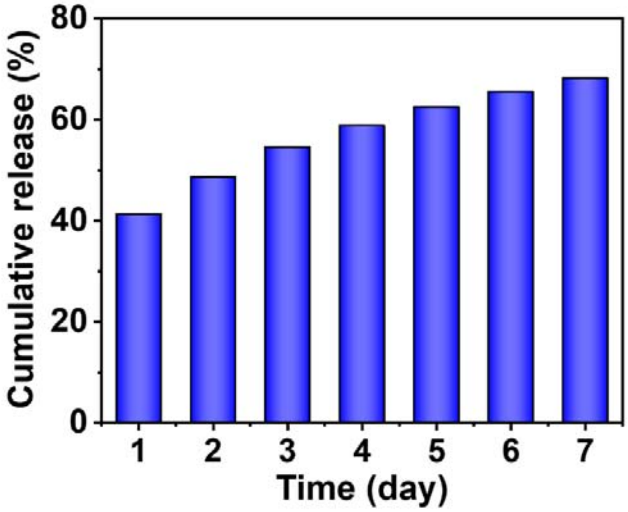

Figure S12. Cumulative release of ISO from βCP-ISO hydrogel.

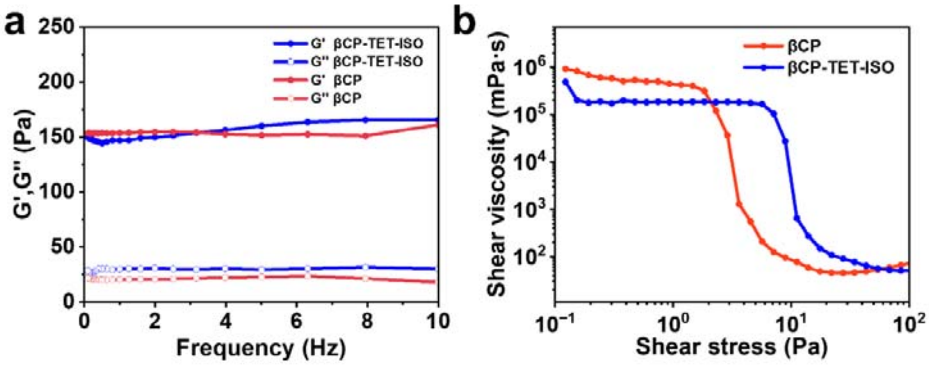

Figure S13. a) Comparison of G′ and G″ between βCP and βCP-TET-ISO hydrogels. b) Shear viscosity-stress curves of βCP and βCP-TET-ISO hydrogels.
